# Simulation and analysis of electro-optic tunable microring resonators in silicon thin film on lithium niobate

**DOI:** 10.1038/s41598-019-42818-2

**Published:** 2019-04-19

**Authors:** Huangpu Han, Bingxi Xiang

**Affiliations:** 1 College of Electric and Electronic Engineering, Zibo Vocational Institute, Zibo, 255314 China; 2 College of New Materials and New Energies, Shenzhen Technology University, Shenzhen, 518118 China; 3 School of Physics, Shandong University, Jinan, 250100 China

**Keywords:** Integrated optics, Silicon photonics

## Abstract

Silicon thin film on lithium niobate combines the advantages of electronic properties of silicon and optical properties of lithium niobate, making it an ideal platform for high-density integrated optics. In this paper, we present an electro-optic tunable microring resonator in silicon thin film on lithium niobate operating at wavelengths of approximately 1.55 μm. The single-mode conditions, optical power distribution, mode profiles, and propagation losses of silicon waveguides are discussed and compared systematically. Quality factor, free spectral range, and bending losses of silicon microring resonators as different radii for different gap sizes between channel and ring waveguides are analyzed in detail. The bending loss and free spectral range decreased with increasing bending radius while the quality factor increased with increasing radius and gap size. The transmission spectrum of microring with radius R = 10 μm was tuned using the electro-optic effect. The key issues affecting the electro-optic effect, such as silicon film thickness and electric field strength, are discussed. This study is helpful for the understanding of microring structures in silicon thin film on lithium niobate, as well as for the fabrication of high-performance and multifunctional photonic integrated devices.

## Introduction

As a semiconductor, silicon (Si) chips demonstrate superb advantages in the integrated electronics industry^1^. Owing to its high refractive index, low optical absorption, mature technology, and semiconductor technology compatibility, Si is a favored medium in the telecommunications wavelength range. However, single crystal silicon does not show a linear electro-optic effect, which limits its application in the field of integrated optics. Lithium niobate (LiNbO_3_, LN) is a widely used electro-optic crystal in integrated optics due to its high Pockel’s coefficients (γ_33_ = 31.2 pm/V)^2,3^. The combination of Si and LN integrates the advantages of matchless electronic properties of Si and excellent nonlinear and electro-optical properties of LN^4,5^. The good confinement and strong guiding of light due to the high-refractive-index contrast between Si and LN enable high-density photonic integrated circuits (PICs). Si thin film on the LN platform has been recently demonstrated^6,7^.

As widely used elements for PICs, microring resonators (MRRs) are attractive because of their ultra-compact size. A wide range of various MRR-based applications for optical communications have been studied, such as add-drop filters^8^, optical modulators^9^, and optical switches^10^. Tunable components have been fabricated using the well-known electro-optic effect^11^. In recent years, several two-dimensional materials have been used to construct polarization-dependent integrated devices, such as graphene, black phosphorus, and rhenium disulfide^12,13^. MRRs in silicon on insulators (SOIs)^14,15^, LN on insulators (LNOIs)^16–18^, and LN on SOIs^19,20^ have been reported in the literature. SOI-based resonators can be used to realize of high-Q resonators, but they do not have true nonlinear optical properties and can only be applied to infrared wavelengths. LN has high Pockel’s coefficients, but LNOI-based resonators have not been integrated into the foundry Si photonics fabrication process popularized over the last decade.

To the best of our knowledge, the simulation and analyses of MRRs in Si thin film on LN have not been reported in the literature to date. In the work described in this paper, therefore, we present a compact and highly linear MRR-based Si thin film bonded to a z-cut LN cladding layer. Height and width measurements of waveguide simulations based on the full-vectorial finite-difference method were performed to investigate the single-mode conditions, optical power distribution in the straight waveguides, mode profile in the bending waveguides, and propagation losses of Si planar waveguides at different LN cladding-layer thicknesses. To obtain a wide free spectral range (FSR) and high quality factor (Q factor), the design parameters, such as ring radii and gap sizes, were studied using 2.5-dimensional variational finite-difference time-domain (varFDTD) simulations. The great potential of Si thin film on LN cladding layer for ultra-compact integrated optics is shown on simulations of electro-optic tunable MRRs at different silicon thicknesses and electric field strengths.

## Results and Discussion

**Figure 1:**
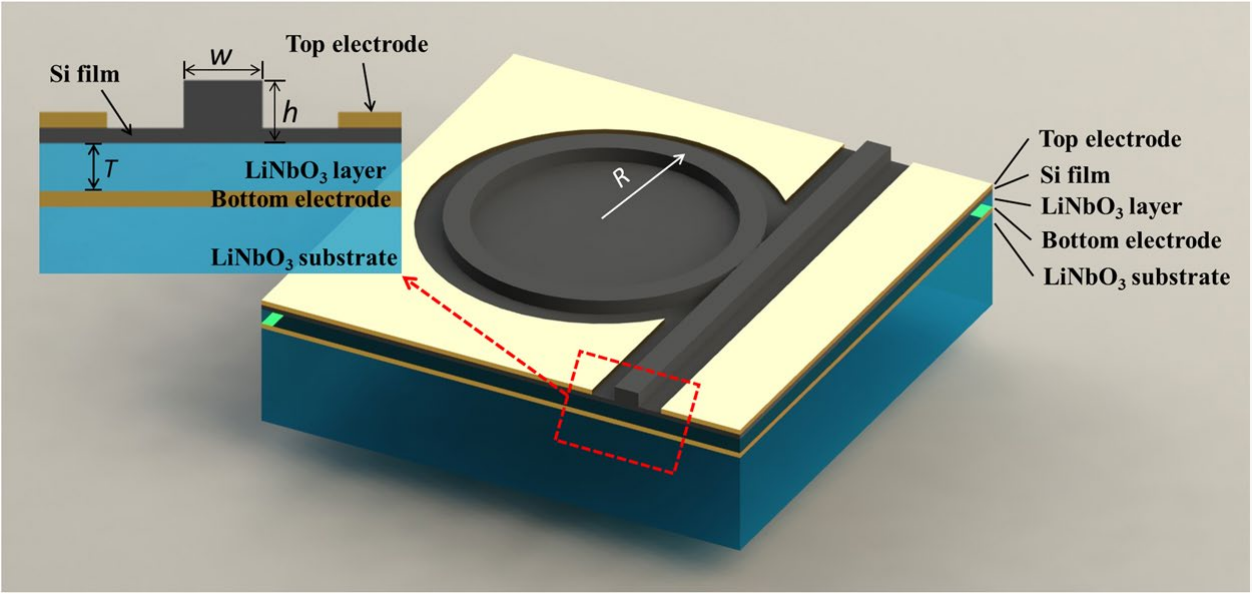
Schematic of Si on LN MRR structure. Inset: Schematic of cross-section of Si on LN waveguide structure.

The device studied in this paper comprised an ion-sliced Si thin film direct-bonded to a z-cut LN layer. The fabrication would be performed at the research center of NANOLN Corporation. The MRR schematic is shown in Fig. 1. The modulator consisted of a ring resonator and a channel made by the Si thin film. If the signal of the channel was on-resonance with the ring, then that signal coupled into the cavity from the channel and coupled out from the cavity into the channel. The entire Si film was not completely etched during the fabrication of the MRRs for two reasons. On one hand, the electro-optic property of the LN layer was not destroyed, while on the other hand the Si film could serve as an optically transparent electrode^19^. Unetched Si with a thickness of 30 nm and top electrodes were used as the upper electrodes in our simulation as shown in the inset of Fig. 1. When adding a bias voltage to Si, a drive electric field can induce a relatively small change of internal carrier distribution; the total amount of carrier was constant, which has a small influence on the refractive index and can be ignored^21,22^. Since the Si layer has good electrical conductively after ion implantation^23^, the vertical profiles between the Si layer and the bottom electrode produced an approximately uniform vertical electric field.

The single-mode conditions in the photonic wire were simulated to prevent signal distortion during transmission. To discuss how light was confined and guided in different waveguide thicknesses and widths, the high-refractive-index difference between Si (n = 3.47 at λ = 1.55 μm)^24^ and LN (n_o_ = 2.21 and n_e_ = 2.13 at λ = 1.55 μm)^25^ required the film thickness *h* and the waveguide width *w* to be smaller than an ultimate value to achieve single-mode operation.

**Figure 2:**
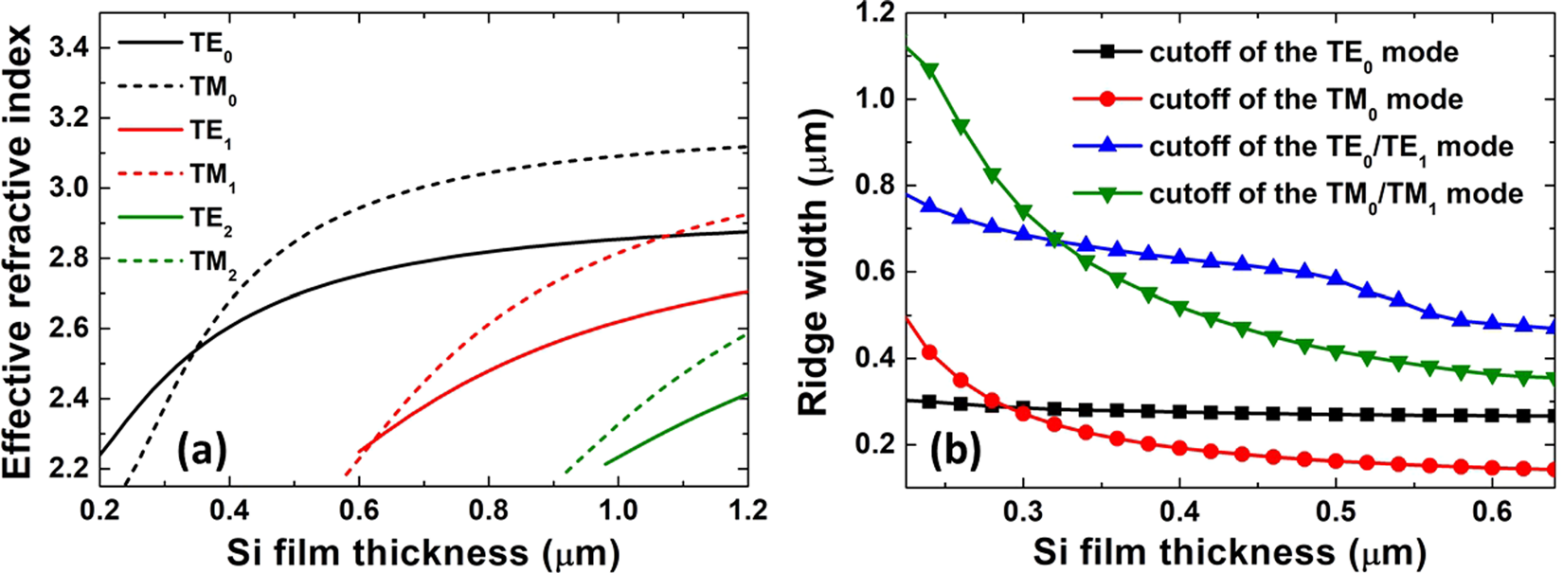
(**a**) Effective index of TE (solid lines) and TM (dashed lines) modes in Si waveguides as a function of film thickness for a waveguide with width *w* = 0.4 μm. (**b**) Cutoff dimensions of TE and TM modes in Si waveguides as a function of width *w* and thickness *h*. Modes were calculated at a wavelength of 1.55 μm.

We calculated the modal curves at wavelength λ = 1.55 μm and Si ridge waveguide width *w* = 0.4 μm. The effective index dependence on the thickness is presented in Fig. 2(a). The first-order mode of the transverse-magnetic (TM)and transverse-electric (TE) modes appeared at Si thicknesses of 0.58 and 0.60 μm, respectively. As the thickness and width of the photonic wire increasing, the effective refractive index increased, and more high-order modes existed. TE_0_ and TM_0_ modes represented the fundamental TE and TM modes. The thickness of Si thin film should be less than this critical value to ensure that only one electric field intensity peak was supported in the vertical direction of the Si thin film. In the following simulation, the Si thicknesses were all selected to be no greater than 0.45 μm.

Figure 2(b) shows the cutoff dimensions of TE and TM modes between the single and multi-mode conditions. The curves represent the boundary at which the corresponding modes vanished. For example, if the Si film thickness was 0.35 μm, to achieve single-mode operation of the TE and TM modes required width *w* = 0.28–0.65 and 0.22–0.60 μm, respectively. To fulfill the single-mode condition, as the width increased, the thickness should decrease. In the following simulation, the widths of Si ridges were all selected as 0.40 μm.

**Figure 3:**
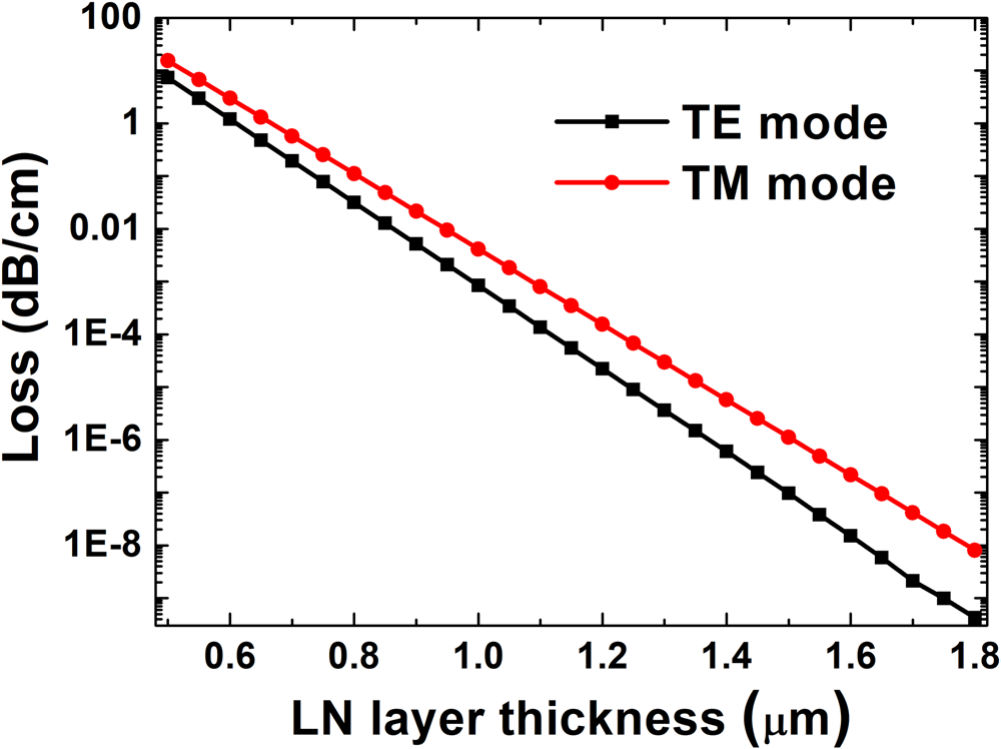
Propagation loss of Si planar waveguide with different LN-layer thicknesses. Modes were calculated at a wavelength of 1.55 μm and a Si thin-film thickness *h* = 0.35 μm.

The LN layer worked as the cladding layer with a thickness T of ultimate value, which was sufficiently thick to prevent field penetration into the bottom electrode. The propagation loss of the Si planar waveguide (thickness h = 0.35 μm) with different LN layer thicknesses at wavelength λ = 1.55 μm is shown in Fig. 3. The propagation loss increased sharply with diminishing LN cladding-layer thickness. The propagation losses of TE modes were less than those of the TM modes at the same LN layer thickness. The Si planar waveguide losses of TE and TM modes were all less than 10^−6^ dB/cm at a LN layer thickness T = 1.5 μm, which was negligible. Therefore, the LN cladding-layer thicknesses were all selected as 1.5 µm in the following simulation.

**Figure 4:**
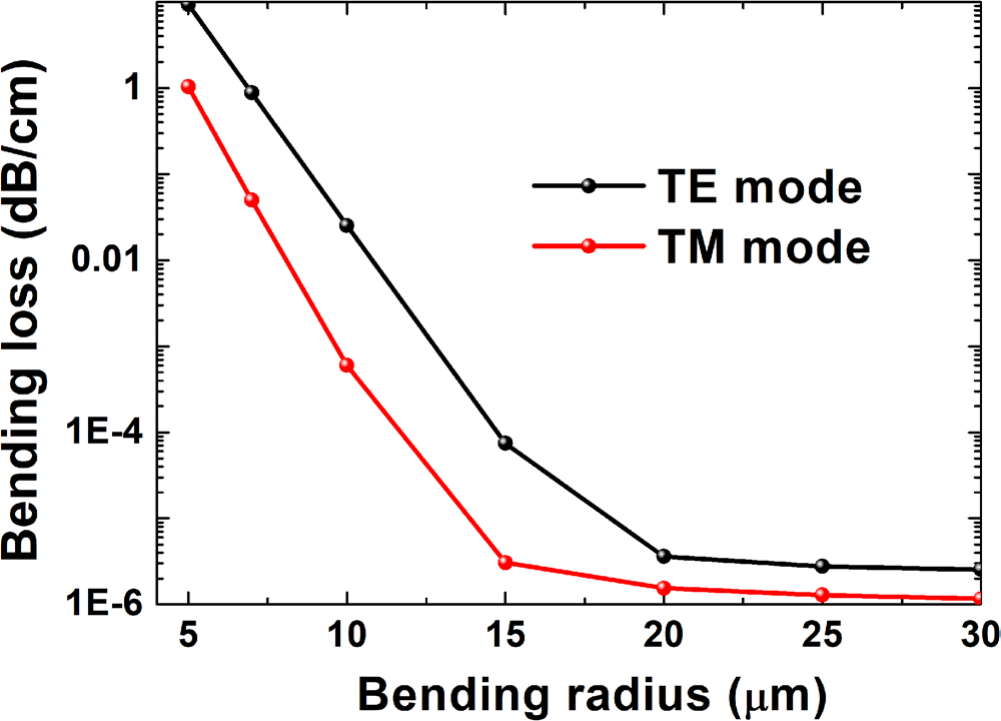
Bending-loss variation as bending radius. Modes were calculated at a wavelength of 1.55 μm.

The bend can lead to radiative losses, which can be measured by using PML boundary conditions to absorb the radiation from the waveguide. The loss values reported by the solver are the net loss of the waveguide, not the loss due to waveguide bend only^26^. The propagation loss is much lower than the bending loss, so we ignore the propagation loss in the calculation. Low bending loss is important to low power consumption devices. Small bending radius was preferred to reduce the dimensions of the photonic devices. Figure 4 shows the relationship between the bending radius and bending loss of a Si ridge waveguide at λ = 1.55 μm. The width and thickness of the Si ridge were fixed as 0.40 and 0.35 μm, respectively. When the radius was smaller than 20 μm, the bending loss increased sharply with decreasing bending radius; when it was larger than 20 μm, it maintained almost unchangeable values. At width *w* = 0.40 μm, height *h* = 0.35 μm, and λ = 1.55 μm, the bending losses of the TM modes were less than those of the TE modes. At a ring radius of 10 μm, the bending losses of the TE and TM modes were 2.537 × 10^−2^ and 6.028 × 10^−4^ dB/cm, respectively. Loss in practical structures arose owing to for the following two factors: (1) the modal mismatch at the interface between Si thin film and LN cladding layer^27^, and (2); on the other hand, the scattering by the residual roughness of the etched surface of the photonic wires^28^. In the experiment, an MRR with a bending loss of 0.1 dB/cm was realized at a bending radius of 80 μm^16^. The propagation losses of the LN waveguides could be further reduced by optimizing the waveguide fabrication process.

A key dimension in MRRs was the gap size separating the ring from the tangential waveguides. The gap sizes determined the input and output couple ratios of the MRRs, which, in turn determined the magnitude of the finesse and the at-resonance transmittance. In the case of a channel coupled to microring, the gap sizes were very small due to the strong optical confinement and the small coupling interaction length^29^.

**Figure 5:**
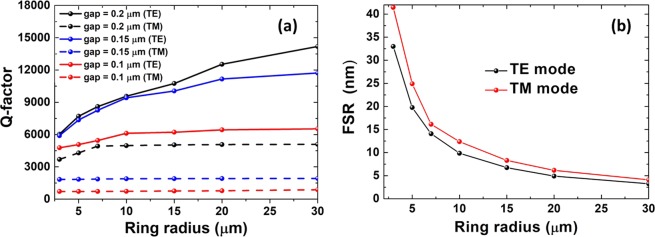
(**a**) Q factor of MRRs for a single-mode waveguide (width *w* = 0.4 μm, thickness *h* = 0.35 μm) as a function of ring radii for different gap sizes. (**b**) FSR of microring resonators at different ring radii. Modes were calculated at λ~1.55 μm.

We calculated the FSR and Q factor for a single-mode waveguide (height *h* = 0.35 μm, width *w* = 0.40 μm) as a function of gap size for different ring radii and assuming TE and TM modes at a light wavelength of approximately *λ* = 1.55 μm. The results are shown in Fig. 5(a,b). The Q factor increased with increasing ring radius and gap size, and the Q factor of the TM mode was smaller than that of the TE mode. When the radius was larger than 10 µm, the Q factor of the TE mode increased slowly while the TM mode maintained almost unchangeable values. However, when the radius was smaller than 10 µm, the Q factor of both TE and TM modes significantly increased with increasing radius. The refractive index contrast of *∆n* ≈ 1.26 (at λ = 1.55 μm) enables the fabrication of rings having a radius as small as R = 10 μm, if other loss sources could be neglected. It was clear that the gap size had a significant effect on device performance. A narrow gap can increase the coupling efficiency between the waveguide and microring, but it proved difficult to fabricate and could cause a reduction in the Q factor. As shown in Fig. 5(b), both TM and TE modes achieved the largest FSR for the ring with the smallest radius, 3 μm, and the FSR increased with increasing ring radius. At width *w* = 0.40 μm, height *h* = 0.35 μm and λ~1.55 μm, the Q factor of the TM mode was smaller than that of the TE mode, and the FSR of the TM mode was greater than that of the TE mode.

**Figure 6:**
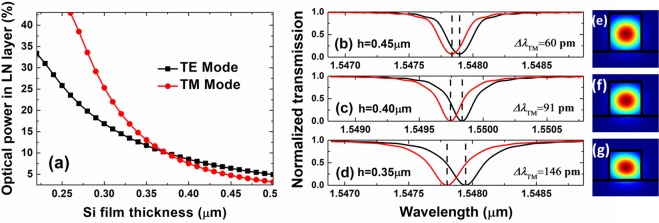
(**a**) Optical power confined in LN layer of TM and TE modes. Modes were calculated at a wavelength of 1.55 μm. (**b**–**d**) Electro-optic shift of resonance curve at different thickness Si thin-film thicknesses. Resonance curves at a wavelength of approximately 1.55 μm (black curve) and corresponding electro-optic-shifted curve in an electric field intensity of 10 V/μm (red curve). (**e**–**g**) Simulated optical power distribution of fundamental TM mode with film width *w* = 0.4 μm, and thicknesses *h* = 0.45, 0.40, and 0.35 μm, respectively, at λ~1.55 μm and microring radius R = 10 μm.

The optical power in the LN layer is the ratio of the optical power in the LN region to the total optical power in all spaces. As shown in Fig. 6(a), the optical power confined in the LN layer of the TM and TE modes increased as the Si film thickness decreased. The reasons could be explained as follows: The Si film thickness was too small to effectively confine all the optical power in the Si thin film, and a small part of the optical power could leak into the LN buffer layer. The light confinement by the Si thin film increased with increasing Si thin-film thickness, and the optical power in the LN layer decreased. At a Si thin-film thickness of 0.35 μm, the values of optical power in the LN layer were 14.45% and 18.46% for the TM and TE modes, respectively.

The electrical tuning of optical resonances was characterized using optical transmission simulations. To observe the electro-optic effect, an electric field considered to be uniform was applied between the top of the Si thin film and the bottom of the electrode by applying a direct current (DC) voltage. Figure 6(b–d) show the shift of the resonance dip at approximately 1.55 μm for TM-polarized light upon applying a DC voltage on the electrodes at different Si thin-film thicknesses. Three shifts of 60, 91, and 146 pm were calculated for an application of an electric field of 10 V/μm at a waveguide width *w* = 0.4 μm, microring radius R = 10 μm, and film thicknesses *h* = 0.45 μm [Fig. 6(b)], 0.40 μm [Fig. 6(c)], and 0.35 μm [Fig. 6(d)], respectively. The electro-optic effect increasing with decreasing Si thin-film thickness because as the thickness of the Si film decreased, the optical power in the LN layer increased, thereby increasing the electro-optic effect. Figure 6(e–g) show the simulated optical power distributions of the fundamental TM mode with film width *w* = 0.4 μm and thicknesses *h* = 0.45, 0.40, and 0.35 μm, respectively, at λ~1.55 μm and microring radius R = 10 μm. As the Si thin-film thickness decreasing, the optical power in the LN layer increased.

**Figure 7:**
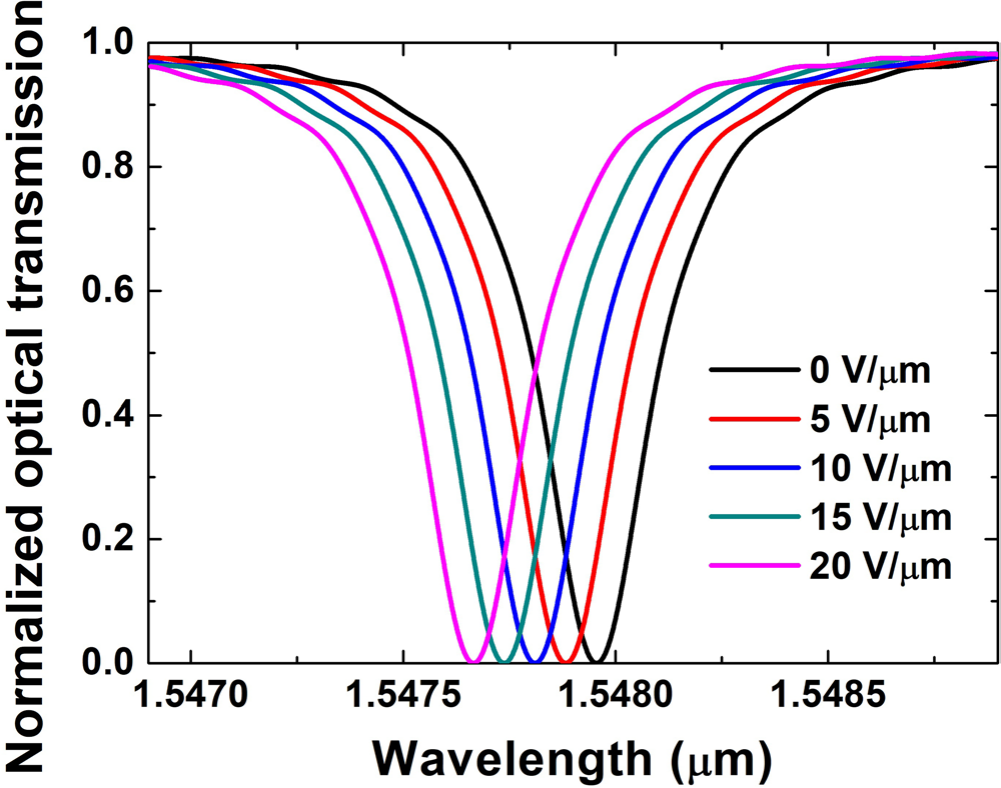
Transmission spectra of wavelength shift due to different electric field intensities in z direction. Calculations refer to TM mode at Si thin-film thickness *h* = 0.35 μm, waveguide width *w* = 0.40 μm, and microring radius R = 10 μm.

Figure 7 shows the simulated TM-mode spectrum as a function of different electric field intensities at a Si film thickness *h* = 0.35 μm, waveguide width *w* = 0.40 μm, and microring radii R = 10 μm. The resonance wavelength shifted increasingly with increasing electric field intensity. For the change in different electric field intensities from 0 to 5, 10, 15, and 20 V/μm, the resonance shifts were 73, 146, 218, and 290 pm respectively indicating was a quasi-linear relationship between resonance-wavelength shift and electric field intensity.

## Conclusions

In conclusion, the Q factor and FSR of MRRs in Si thin film on z-LN were calculated by varFDTD. Using the full-vectorial finite-difference method, single-mode conditions, optical power distribution, mode profiles, and propagation losses of Si waveguides were calculated. The optical power in the LN cladding-layer increased with decreasing Si thin-film thickness. The propagation losses of Si planar waveguides decreased with increasing LN layer thickness. The thickness of the Si film, width of the ridge waveguide, and thickness of the LN layer were optimized to 0.35, 0.40, and 1.5 μm, respectively. The effects of ring radius and gap size on the Q factor, FSR, and bending loss were simulated and discussed. The Q factor increased with increasing bending radius and gap size, while FSR and bending loss decreased with increasing bending radius. As it is a very important aspect of practical applications, we described electro-optic tunable MRRs. The relationships between electric field strength, Si thin-film thickness, and electro-optic effect were studied, and simulation results show that the resonance-wavelength drift of the electro-optic micro-resonator in Si thin film increased with increasing electric field strength and decreased with increasing Si thin-film thickness.

## Methods

The full-vectorial finite-difference method was used to calculate the single-mode conditions, optical power distribution in the straight waveguides, mode profile in the bending waveguides, and propagation losses of Si planar waveguides at different LN cladding-layer thicknesses in the simulation. The operating principle of this method is as follows. The finite-difference algorithm was used for meshing the waveguide geometry, and had the ability to accommodate arbitrary waveguide structure. Once the structure was meshed, Maxwell’s equations were then formulated into a matrix eigenvalue problem and solved using sparse matrix techniques to obtain the effective index and mode profiles of the waveguide modes^26^.

The varFDTD method with perfectly-matched-layers (PML) boundary conditions was used to calculate the Q factor and FSR in the simulation. The varFDTD method was a direct time and space solution for solving Maxwell’s equations in complex geometries. By performing Fourier transforms, the Poynting vector, normalized transmission, and far-field projections could be obtained. PML boundaries could absorb electromagnetic energy incident upon them, allowing radiation to propagate out of the computational area without interfering with the field inside.
